# Toxicological effects of nanoselenium in animals

**DOI:** 10.1186/s40104-022-00722-2

**Published:** 2022-06-17

**Authors:** Iqra Bano, Sylvie Skalickova, Safia Arbab, Lenka Urbankova, Pavel Horky

**Affiliations:** 1 Department of Physiology and Biochemistry, Faculty of Bioscience, Shaheed Benazir Bhutto University of Veterinary & Animal Sciences, Sakrand, 67210 Pakistan; 2 Department of Animal Nutrition and Forage Production, Mendel University in Brno, Zemedelska 1, CZ-613 00 Brno, Czech Republic; 3 Key Laboratory of Veterinary Pharmaceutical Development, Ministry of Agriculture, Lanzhou Institute of Husbandry and Pharmaceutical Sciences, Chinese Academy of Agricultural Sciences, Lanzhou, 730050 China

**Keywords:** Nanoparticles, Organism, Selenium, Toxicity, Trace minerals

## Abstract

The productivity and sustainability of livestock production systems are heavily influenced by animal nutrition. To maintain homeostatic balance in the body of the animal at different phases of life, the percentage of organically active minerals in livestock feed must be optimized. Selenium (Se) is a crucial trace mineral that is required for the maintenance of many functions of the body. Se nanoparticles (SeNPs) attracted considerable interest from researchers for a variety of applications a decade ago, owing to their extraordinary properties. SeNPs offer significant advantages over larger-sized materials, by having a comparatively wider surface area, increased surface energy, and high volume. Despite its benefits, SeNP also has toxic effects, therefore safety concerns must be taken for a successful application. The toxicological effects of SeNPs in animals are characterized by weight loss, and increased mortality rate. A safe-by-strategy to certify animal, human and environmental safety will contribute to an early diagnosis of all risks associated with SeNPs. This review is aimed at describing the beneficial uses and potential toxicity of SeNPs in various animals. It will also serve as a summary of different levels of SeNPs which should be added in the feed of animals for better performance.

## Introduction

**Fig. 1:**
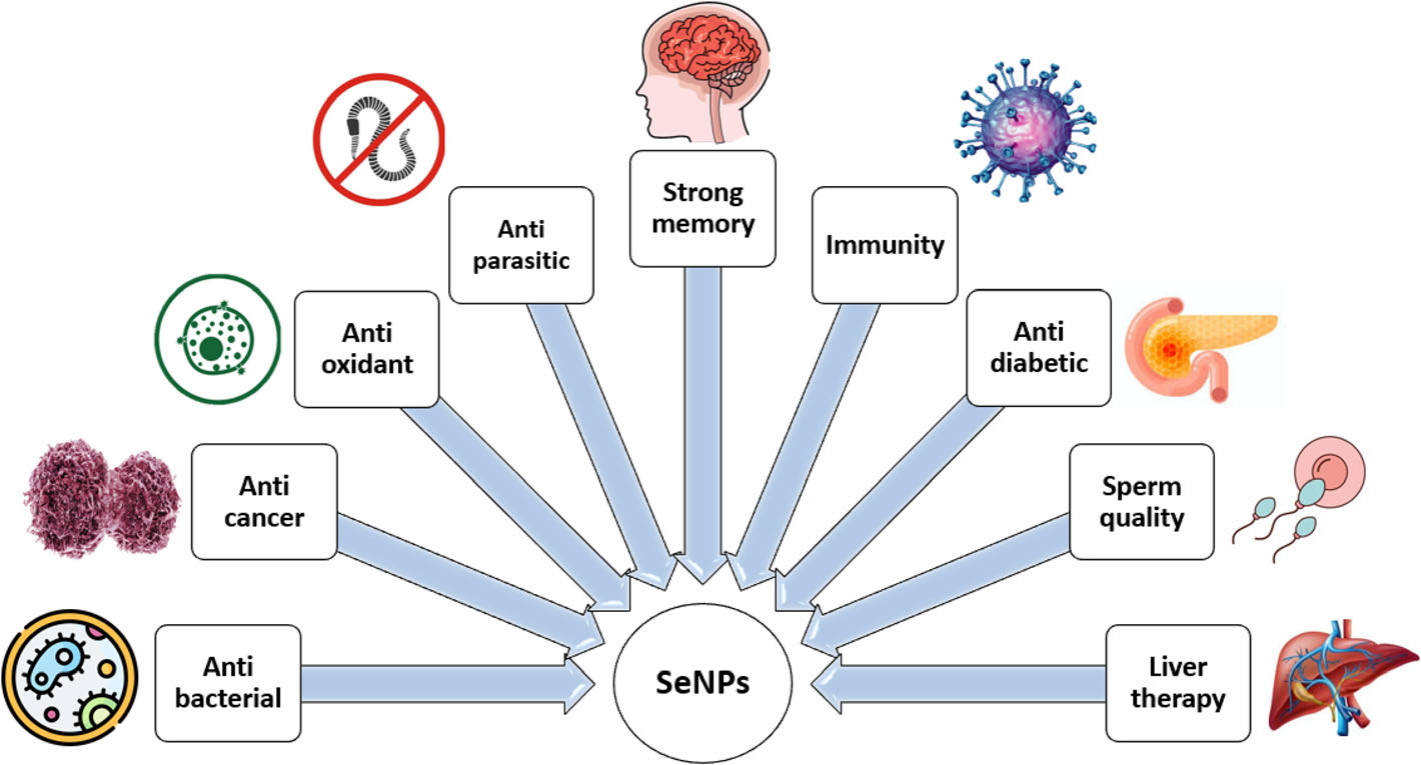
The representation of some important biological prospects and effects of SeNPs

Recent years have witnessed a growing academic interest in nanotechnology development agriculture [1]. Inorganic nanoparticles (NPs) are fast becoming a prospective instrument in animal feed. They promise an improvement of properties of traditional mineral elements, through their biologic efficiency [2], bioavailability, or antimicrobial effects [3]. NPs are recognized as particles less than 100 nm in diameter, prepared by synthetic or biological ways. Previous studies have observed that NPs can maintain excellent bioavailability and decreased toxicity compared to inorganic and organic formulae of trace minerals [4]. The most frequently discussed mineral compound is selenium (Se) due to its narrow relationship between toxicity and necessity for organisms [5]. The biological efficacy of Se is based on its integration into the active center of 25 selenoproteins (SeLPs) [6] . Organic forms of Se and specific salts have been studied for many years [7], but elemental Se nanoparticles (SeNPs) have recently received a great deal of attention as a potential source of this vital component [8]. Figure 1 below illustrates the biological proceptivity and effects of SeNPs which have been experimentally observed.

A few studies have shown that SeNPs have a lower toxic potency than dissolved ionic Se species, which is a promising finding [9]. The evidence suggests that Se from NPs becomes less bioavailable to some extent [10]. Furthermore, the toxicity of SeNPs could be reduced through green synthesis or modification. Numerous experiments of SeNPs toxicity have been conducted in animals, but proper knowledge about the toxicological effects of SeNPs is insufficient. This review is aimed to evaluate the updated information regarding the toxicological effects of SeNPs in animals.

## Toxicity by selenium intake

Se poisoning is a threat in geographical areas with a high abundance of Se in the environment. Continuous intake of water or feed rich in Se can lead to its accumulation and selenosis in the body [11]. Acute Se poisoning of grazing animals occurs as a result of the consumption of a large number of accumulator plants with a high concentration of in a short period of time. For example, seleniferous plants include prince’s plume, astragalus and woody asters [12]. According to scientific evidence, all species of animals are vulnerable to Se toxicosis. Symptoms of Se poisoning in mammals vary widely and include nail abnormalities and loss of hair and wool [13], weakness, vomiting, diarrhea, tiredness, reduced cognitive function, lethargy, immobility, fatigue, weight loss, itchy skin and mucous membrane irritation [14]. Individuals who have the condition may experience lateral sclerosis as well as irritation in the pharynx and bronchial tubes, and may be recognized by a garlic smell on their breath and in their sweat [11].

On the biochemical level, Se toxicosis includes splenomegaly, anemia, liver damage, and elevated ratios of bilirubin respectively [15]. During the first 24 h after acute poisoning, Se concentrations in the kidneys and liver drop by 80% from peak levels, according to animal studies [14]. An examination of Se poisoning in domestic animals has shown that there was an increase in the rate of conception and the fetal resorption in bovine, sheep, and horses fed naturally organic Se-containing diets with 25–50 mg Se/kg [16] . Poisoning can also occur in swine, fish, and other grain-consuming species raised on seleniferous soils or, more often, due to errors in feed formulation [17].

Acute Se toxicity could lead to brain disorders, changes in mental status, gastrointestinal symptoms, breathing difficulty, hepatocellular necrosis, kidney failure, heart attacks, and other cardiac disorders. Some research has shown Se intoxication can delay the growth of animals [11]. Younger animals are more sensitive to Se poisoning and the chemical forms may lead to differences in toxicity [18]. In addition to mammals, Se has a wide range of harmful consequences in birds, and the onset of toxicity varies from several hours to days [19]. The toxic effects in avian species include mortality, decreased growth, histopathological abnormalities, and changes in hepatic glutathione (GSH) metabolism [20].

### General mechanism of se toxicity

It has been shown that Se toxicity greatly depends on its form. Generally, organic Se compounds are known to be less hazardous to cells than selenite, when investigated both in vitro and in vivo [18]. Se species metabolize by several pathways into different chemical forms, or they are incorporated into selenoproteins. In addition, due to the chemical similarity of Se with Sulphur, Se can be involved in the biochemical pathways of thiol compounds. Scientific evidence shows Se can spontaneously interact with glutathione to form Se^0^, glutathiolseleol (GS-Se), selenodiglutathione (GS-Se-SG), hydrogen selenide (H_2_Se) [21] and selenotrisulfides. Selenotrisulfides can react with other thiols to produce superoxide and hydrogen peroxide, both of which are toxic [22]. In addition, Se exposure promotes redox imbalance and the production of reactive oxygen species in eucaryotic cells [11].

#### Mechanism of se induced genotoxicity

The genotoxicity of Se has been studied extensively. This genotoxicity occurs when an excess of ROS is present in cells and reacts with cellular components. This causes base lesions as well as breakage of deoxyribose nucleic acid (DNA) strands via its reaction with both deoxyribose sugars and the nucleobases of DNA. In addition, ROS oxidizes DNA, and Se interferes with DNA repair and transcriptional regulation, posing a threat to the stability of genetic information. Further, Se also interacts with some DNA repair proteins that contain functional zinc (Zn) finger motifs, which are associated with signaling pathways, such as DNA repair peptides, and DNA protein-protein interaction factors. Se can also interact with metallothionein and cause the release of Zn, which can affect DNA-binding capacity as well as genome stability [23]. Several authors have proposed that Se causes genotoxicity by communicating with thiol groups by these means. On the other hand, it was discovered that the number of dicentric chromosomes is roughly 2 times higher in Se-plus radiation exposure treatment compared to the control group [24]. In addition, Se causes genotoxicity by interfering with the ataxia-telangiectasia mutated gene and protein 53 expressions in the body. It have been shown that mice treated with methylselenic acid and methyl selenocysteine in ten days treatment delaying in the disease’s progression by increasing apoptosis and decreasing proliferation was observed [21].

#### Mechanism of se induced cytotoxicity

Many researchers have investigated the cytotoxicity of Se, which causes irreversible changes in cells through a variety of mechanisms. It has been found when cells are exposed to Se, the production of ROS can increase. Also, Se induces the production of ROS as a result of the selenide (Se^2−^) reaction with thiol groups [25]. Excess ROS damages not only lipids and proteins but also mitochondrial membrane potential. According to one study, ROS-induced oxidative stress results from the activation of the mitochondrial apoptotic pathway [26]. It has long been known that ROS causes cytotoxicity by activating c-Jun N-terminal kinases (JNK), a subgroup of mitogen-activated protein kinases that regulates a wide range of cellular functions including cell proliferation, differentiation, and apoptosis. ROS can stimulate the JNK-mediated tumor necrosis factor [27]. ROS can also act as signal transduction pathway modulators, which can impact a variety of biological processes such as cell growth, apoptosis, and cell adhesion, among others [28]. It has been discovered that Se, a constituent of SelPs, seems to have a close relationship with redox potential, which can cause cytotoxicity by altering thioredoxin reductase (TrxR). This altered TrxR, when combined with thioredoxin (Trx), forms a potent dithiol-disulphide oxidoreductase system [29]. In addition to binding to signaling molecules (including apoptosis signal-regulating kinase-1 and Trx interacting protein), the system can also regulate cell growth by interacting with the cells’ growth and survival mechanisms. Glutaredoxin proteins, which are redox-active proteins, have been associated with susceptibility to Se cytotoxicity by limiting intracellular cystine levels, according to another research group [30]. As Se can modulate cell signaling pathways through the use of a thiol redox system, it causes cytotoxicity through the production of ROS, as well as by affecting the expression of correlating genes and proteins [31].

## The toxic effects of SeNPs

Various animal species have different sensitivities to the effects of Se and SeNPs. The toxicity of nanoparticles has mainly been studied in aquaculture due to these species’ sensitivity to water pollutants. The toxicity of SeNPs in aquaculture has been well documented and reviewed in recent studies. According to a review article by Abbas et al., it has been implied that the nanoforms of Se are particularly toxic compared to inorganic Se salts [32]. This finding is alarming in that most of the nanomaterials used, including SeNPs, accumulate in the environment and can reach fish that subsequently bioaccumulate SeNPs in large quantities. In contradiction, however, it has also been reported that the SeNPs can increase the productivity of aquatic animals and improve their health in controlled experiments [33]. Similar to the effect in mammals, the toxicological effect in fish depends on the dose, the chemistry of the SeNPs, and the exposure time. Regarding the toxicity of SeNPs, this section reviews the literature on toxicological studies of SeNPs. The findings are summarized in Table 1. To compare SeNPs effect on the mammalian organisms the chemoprotective studies of SeNPs are included in the Table 2. It is apparent, the SeNPs effects on organism are greater than inorganic Se forms. In addition, the impact of Se on the health status depends on individual need to create antioxidant defence. Otherwise, an excess of Se leads to its toxicity. The toxicity of SeNPs has been thought to be related to Se toxicity in general. At higher concentration, both Se and SeNPs have pro-oxidative properties leading to ROS production [34]. This effect could be enhanced by the bioaccumulation effect in several tissues where the liver is most sensitive.

**Table 1:** Summary of toxicologic studies of SeNPs in various mammalian species

Compare study	Animal species	Size, nm	Modification	Dose	Exposed time, d	Effects	LD50	Ref
	Mice	35		0.1 mg Se/kg diet	45	SeNPs-M showed↑ Se retention and the levels of glutathione peroxidase, superoxide dismutase and catalase	72 mg/kg	[45]
	Mice	20		200 μg Se/kg BW/d	90	Under the safe dose (0.75–7.5 mg/kg), oral administration of PTR-SeNPs dramatically inhibited the growth of cancer in a tumor-bearing nude mouse mode	20 mg/kg	[46]
	Mice	40–55		2 mg Se/kg BW/d	28	SeNPs, caused↓ bone marrow cell death and prevented DNA damage, compared to other forms of selenium		[47]
	Mice	20		0.5, 5, and 50 mg Se/kg diet	14	Toxicity ↑ when inorganic Se was applied than after subacute application of Sel-Plex, nanoSe, or LactoMicroSe		[48]
	Mice	70–90		1 and 4 mg Se/kg	28	Nano-selenium at low dose (1 mg/kg) exhibited antioxidant effects in the liver compared to the high dose (4 mg/kg) of SeNPs and sodium selenite (1 and 4 mg/kg)	113.87 mg/kg	[49]
	Mice	50	Chitosan	10.5 g Se/kg	45	Acute fetal test showed SeNPs-C/C was safer than selenite, with a median lethal dose (LD50) of approximately 4-fold to 11-fold of that of selenite	8.8 mg/kg	[50]
Na_2_SeO_3_	Mice	5		2, 4 and 6 mg/kg BW	15	Selenite and SeNPs completely and partially suppressed mice growth respectively. Abnormal liver function was more pronounced with selenite treatment than SeNPs	15.7 mg/kg	[51]
SeMetCys	Mice	20–60		10 mg Se/kg	7	↓Body growth, ireversible changes by SeMSC, reversible changes by SeNPs in liver; ↑ serum ALT and LDH in SeMSC compared to SeNPs and ctrl. ↑ GST activity in SeNPs group compared to SeMSC and ctrl; ↓ T-AOC in SeMSC group, not in SeNPs group	SeMSC 14.6 mg Se/kg and SeNPs 92.1 mg Se/kg	[39]
SeMet	Mice	20–60		10 mg Se/kg	7	↑Gpx and thioredoxin reductase, ↓toxicity as indicated by median lethal dose, acute liver injury, and short-term toxicity by SeNPs	27.0 mg/kg	[52]
SeO_2_	Mice	80–220	Green synthetized via Bacillus sp.	2.5, 5, 10, **20** mg/kg BW	14	↓ Body weight, ↑ AST, ALT, ALP, Cr, Chol, TG, TB and worsed hematological parameters in total blood at the dose of 20 mg/kg	SeO2–7.3 mg/kg SeNPs 198.1 mg/kg	[53]
	Rats	78.88		2, **4**, and **8** mg Se/kg BW	14	↓ Antioxidant capacity in serum, liver, heart; ↓ expression of GPx-1 and GPx-4 in liver; ↑MDA in liver		[54]
	Rats	79.88		0.2, 0.4, 0.8, **2.0, 4.0**, or **8.0** mg Se/kg BW	14	↓ Body weight, ↑ ALP, SAST, CHol, ↑ liver weight; ↓ thymus weight; ↑ Apoptotic cells count in liver		[37]
	Rats	4.6, 24.5	κ-carrageenan-capped SeNPs	500 μg/kg BW	10	↓ Count of astroglial cells in brain; ↑ Se accumulation in liver, kidneys, brain in 4.6 nm SeNPs treated group; − changes in internal organs and glands		[37]
Na_2_SeO_3_	Rats	100–150	Green synthetized via potatoe extract, PEG coated	5, 10, 15 μg/kg	21	Organ weight in SeNPs groups; ↓ decreased weight of internal organs in sodium selenite group; no differences in heamatological parameters in sodium selenite group X markable changes in SeNPs group compared to ctrl; sodium selenite negatively affected; histopathology of liver, but not SeNPs; ↓ concentration of Se in breast milk in SeNPs compared to sodium selenite and ctrl group		[55]
Na_2_SeO_3_	Rats	20		0.05, **0.5**, or **4** mg Se/kg BW	28	↓ Body weight; − neurotransmitters, hematological parameters, histology of liver		[35]
Na_2_SeO_3_	Rats	80	PVA modified	1.2 mg Se/kg	30	↓ GSH in liver for Se, SeNPs groups; ↑ GSSG in liver for Se, SeNPs groups; higher retention of Se in group of SeNPs compared to Se group in blood		[56]
	Rats	79.88		0.2, 0.4, 0.8 mg Se/kg BW	14	The supranutritional ↑ sperm motility and movement parameters, The nonlethal levels of 4.0 and 8.0 mg Se/kg BW ↓ testisweight, sperm concentration, and motility and also caused histopathological injury of testisand epididymis tissues to various degrees		[57]
	Rats	100		0.5, 1.5, 3.0 and 5.0 mg Se/kg	28	Histopathological examination showed damage to the liver parenchyma and intestinal epithelium, ↓ ALT activity	7 mg/kg	[58]
Na_2_SeO_3_	Rats			10, 18 mg/kg	10	CK, CK-MB and LDH levels of Group IV ↑ other groups on both the 2nd and 10th days. In Groups II and III, this serum level decreased, and vitamin B_12_ ↑	10 mg/kg	[59]
	Rats	5–100		2, 3, 4 and 5 ppm	91	The toxicity was ↑more pronounced in the selenite and high-selenium protein groups than the Nano-Se group	113 mg/kg	[60]
Na_2_SeO_3_	Rats	20–60		0.0096 and 0.1 ppm	14	SeNPs has a 7-fold lower acute toxicity than sodium selenite in mice (LD50 113 and 15 mg Se/kg body weight respectively	15.7 mg/kg	[61]
Na_2_SeO_3_	Rabbits			0.3 mg/kg BW	42	− Chol, TG, TP, Glu, ALT, AST, ↑ GPx mRNA expression, TAOC		
Na_2_SeO_3_	Chickens	100	Green synthetized	0.3 mg Se/kg diet	42	− Serum glucose, cholesterol, lipoprotein, thyroid hormone, and liver function levels and biomarkers of kidney function; ↓ lowest relative weight of the liver; ↑ otal protein in serum		[62]
	Chickens	60		0.15, 0.30, 0.60 and 1.20 mg/kg/d	49	Se in serum, liver and breast muscle ↑, magnitude of increase was substantially ↑ when Nano Se was fed	113.0 mg/kg	[63]
SeYeast, SeMet	Chickens			0.1 and 0.3 mg/kg diet	42	SeNPs improved yellowness, redness and meat quality, NS and organic sources of Se resulted in better meat quality		[64]
	Chickens	100		0.3, 0.9 and 1.5 ppm	29	inorganic Se caused↓bioavailability in breast and duodenum tissue and↑ accumulation in organs involved in detoxification compared to organic selenium SeNPs		[65]
	Chickens	200		0.15, 0.30, 0.45 ppm	32	SeHME showed ↑ expression of GPx-4 in the livers and SelW in the spleens compared with SeS treatment		[66]
	Chickens	100		0.3, 0.9 and 1.5 ppm	29	Inorganic Se leads↓ bioavailability in breast and duodenum tissue and ↑ accumulation in organs involved in detoxification processes as compared to organic Se and SeNPs		[65]
	Sheeps	40		5 mg Se/kg BW	30	HB, RBCs, and PCV in Nano-Se ↓, SLD, GOT, CTT and AP in Nano-Se group was↑. Levels of IgG, IgM, IgA, IL-2,TNF-α in NanoSe group were↓ than those of the control.		[67]
SeMet, Na_2_SeO_3_	Piglets	28–59		0.3 mg Se/kg diet	28	↑ Glutathion peroxidasis, expression of selenoprotein W (SELW), GPx1, and GPx3 in the liver		[68]
	Pigs	100		0.5 mg Se/kg diet	45	− Performance; ↑ concentration Se in muscle, T-AOC, GPx, SOD, CAT; ↓ MDA		[69]
SeYeast	Sheep			4 mg/kg	25	Ruminal pH, ammonia N concentration, molar proportion of propionate, ratio of acetate to propionate ↓and total ruminal VFA concentration was ↑ with NS and YS		[70]
Na_2_SeO_3_	Cows	100		0.3 mg Se/kg diet	30	−Matter intake, milk yield and composition; ↑ plasma Se levels and GPx; ↓ mRNA expression levels of glutathione peroxidase 1, 2 and 4; thioredoxin reductase 2 and 3; and selenoproteins W, T, K and F		[71]

**Table 2:** Summary of original research articles focusing on the chemoprotective effect of SeNPs on various mammalian species

Compare study	Animal species	Injury	Size, nm	Modification	Dose	Exposed time, d	Effects	Ref.
Na_2_SeO_3_	Mice	Inducet atherosclerosis	23, 40, 86		50 μg Se/kg BW	24	↓ Atherosclerotic lesions; ↑ oxidative stress; ↓ GPx; ↑ hyperlipidemia in liver (observed changes were significantly higher in sodium selenite group; moreover SeNPs at the size of 40 nm showed highest negative impact on animal health)	[44]
Na_2_SeO_3_	Mice	Alcohol-induced gastric mucosal injury	60	Chitosan	1.58–5 mg/kg BW	30	LD50 sodim selenite: 8.8 mg/kg BW; LD50 SeNPs 73.2 mg/kg BW; − body weight, viscera indexes of heart, liver, spleen and kidney (not in liver); SeNPs showed gastroprotective properties; ↑ SOD, GSH-Px and CAT in gastric mucosa in SeNPs treated groups	[72]
	Mice	Oxidative stress	50	Chitosan	10.5 mg/kg	60	Acute fetal test showed SeNPs-C/C was safer than selenite, with a median lethal dose (LD50) of approximately 4-fold to 11-fold of that of selenite	[50]
Na_2_SeO_3_	Mice	0, 2, and 8 Gy gamma irradiation.	20–50		0.1 mg/kg	14	Selenium nanoparticles as an emerging potent antioxidant agent can protect against irradiation induced nephropathy	[73]
	Mice	oxidative stress	200	Melatonin modified SeNPs	10 mg/kg	10	MTse protects against hepatocellular damage than a similar dose of melatonin (10 mg/kg) or selenium (0.1 mg/kg) alone	[74]
	Mice	Gentamyin induced nephrptoxicity	30–100		2 mg/kg BW	10	SeNPs are potent antioxidant candidate against GM-induced oxidative kidney toxicity and hematoxicity in mice.	[75]
	Mice	Eimeriosis-induced inflammation	5–50		0.5 mg/kg	5	SeNPs were able to regulate the gene expression of mucin 2, interleukin 1β, interleukin 6, interferon-γ, and tumor necrosis factor α in the jejunum of mice infected with *E. papillata*	[76]
	Mice	Hepatocytes exposed to Gamma radiation	50–200		0.10 mg/kg	14	Selenium nanoparticles bear a more potent antioxidant effect in comparison with selenium selenite and can effectively protect the liver cell against Gamma radiation at a dose of 8.00 Gy	[77]
	Mice	Cellular damage in thyriod by chromium	3–20		0.5 mg/kg	5	Se nanoparticles have a protective effect on K_2_Cr_2_O_7_-induced thyroid damage, as a result of correcting the free T_3_ and T_4_ levels and GSH, catalase, SOD, and MDA compared to the K_2_Cr_2_O_7_-treated group.	[78]
	Rats	Deltamethrin induced effects on sperm characteristics	100–200		0.5 mg/kg BW	60	↑ Sperm count, motility and viability; ↑ body weight; − testosterone; ↑ GPx, TAC; ↓ MDA	[79]
Na_2_SeO_3_	Rats	Glycerol-induced acute kidney injury	129.3	Green synthesis with lycopene	0.5 mg/kg	14	↑ Renal biochemical profile, GPx, ↓ MDA; ↑ expression of *IL-1β*, *IL-6*, and *TNF-α* genes; ↓ caspase-3, Bax, and cyt-c	[80]
	Rats	Chloride-induced hepatorenal toxicity	100		0.4 mg/kg BW	21	− Creatinine levels; ↓ MDA; ↑ GSH, SOD in renal tissue; ↑ expression Bcl-2 (antiapoptotic protein); ↓ caspase-3 activity	[81]
Na_2_SeO_3_	Rats	Paracetamole induced toxicity	40		0.5 and 1 mg/kg	30	− ALP, AST, ALT, LDH, GPx in Se and SeNPs groups; protective effect of Se and SeNPs against paracetamol	[82]
	Rats	Tert butyl hydroperoxid induced oxidative stress	42		0.3 mg/kg BW	35	↓ SOD in liver in SeNPs and t-BHP treated rats compared to ctrl; ↑ GPx, CAT in liver in SeNPs groups; − liver enzymes among treated groups compared to ctrl	[83]
	Rats	Streptozocin induced diabetes	20–80		0.1, 0.2 and 0.4 mg/kg BW	28	↓ Blood sugar, albumine in blood; ↓ creatinin, urea	[84]
Na_2_SeO_3_	Rats	Bisphenol-induced reproductive toxicity	20–60		2 and 3 mg/kg BW	70	↑ Antioxidant status; ↓ MDA; ↑ restoration of testicular tissue; ↓ expression of mRNA of *COX-2*; ↑ expression of mRNA of *ER-2*; ↓ DNA fragmentation compared to ctrl and sodium selenite group	[85]
	Rats	Induced bone toxicity	40–90		0.25, 0.5, 1 mg/kg/d	28	↑ Bone density and biochemical markers of bone resorption	[86]
	Rats	Neurobehavioral abnormalities and oxidative stress caused by 1-methyl-4-phenyl-1,2,3,6-tetrahydropyridine		Glycine	0.05 and 0.1 mg/kg BW	30	↑ Rat’s behaviour and number of TH^+^ neurons; ↓ MDA; ↑ SOD and GSH-PX	[87]
	Rats	Oxidative injury	50	Chitosan	280 mg/kg	30	↑ Testicular function; ↑ testosterone levels, ameliorating testicular tissue; ↓markers of oxidative stress in male rats	[88]
	Rats	Renal injury	68–122		0.1 mg/kg	14	↑ Kidney relative weight; ↑ serum urea, creatinine, Kim-1, and renal malondialdehyde, nitric oxide, TNF-α, IL-1β, cytochrome c, Bax, and caspase-3 levels	[89]
	Rats	ACR-induced injury	25–51	Chitosan	0.2 mg/kg/d	60	Ch-SeNPs (0.2 mg/kg/d) displayed more protection against ACR-induced damages comparing to Na_2_SeO_3_	[90]
	Rats	Reproductive toxicity			0.5 mg/kg	60	SeNPs improved DLM-induced negative effects on sperm characteristics, testosterone, and antioxidant biomarkers, as well as behavioral and histopathological alterations. The SeNPs treated group showed improved semen parameters, antioxidant status, and sexual performance	[79]
	Rats	Streptozotocin STZ-induced diabetes	10–80		0.1 mg/kg	28	SeNPs increased the glutathione content and antioxidant enzyme activities in testicular tissues. Moreover, microscopic analysis proved that SeNPs are able to prevent histological damage inthe testes of STZ-diabetic rats	[91]
	Rats	Diabetic nephropathy during pregnancy			2.5 mg/kg	42	SeNPs significantly reduced the rate of urination, accelerated the start of gestation, and increased the percentage of successful pregnancy in females with DM	[92]
	Rats	Carbon tetrachloride-induced toxic damage of liver	15–27		0.1 mg/kg	14	A high dose of SeNPsto rats with toxic liver damage decreases the concentration of lipid peroxidation products in the blood and normalizes the level of liver enzymes at a time of the damage of the urinary system	[93]
	Rats	Carbon tetrachloride-induced hepatotoxicity	200–300		2.5 mg/kg	21	SeNPs pretreatment significantly improved the level of AST, urea, creatinine, MDA, LDH, and GSH in the CCl_4_ -injected rats towards the control levels	[94]
	Rats	Cypermethrin-induced neurotoxicity	100		2.5 mg/kg	21	SeNPs increased levels of GABA and glutathione; on the other hand, it significantly prevented the rise in the levels of MDA, TNF-α and IL-1β	[95]
	Rats	Nephropathy			5 mg/kg	30	Reduced glutathione and malondialdehyde levels in tissue samples were correctly modulated in the pups from N.P.s treated diabetic mothers.	[96]
	Rats	Cadmium chloride (CdCl_2_)-induced neuro- and nephrotoxicity	3–5, 10–20		0.5 mg/ kg	56	SeNPs significantly ↓ CdCl_2_-induced elevation of serum kidney and brain damage biomarkers; lipid peroxidation; the percent of DNA fragmentation and nearly normalized the activity of acetylcholinesterase (AchE) and↑ activity and expression of antioxidant biomarkers	[97]
	Rats	Brain oxidative damage			0.1 mg/kg	45	Enhanced brain antioxidant status and lower AChE activity and oxidative-inflammatory stress biomarkers. A significant downregulation of caspase 3 and upregulation of parvalbumin and Nrf2 protein expressions was observed in treated groups	[98]
	Rats	MEL-induced renal function impairments	3.3–17	Green synthesis	0.5 mg/kg	28	MEL-induced nephropathic alterations represented by a significant increase in serum creatinine, urea, blood urea nitrogen (BUN), renal TNFα, oxidative stress-related indices	[99]
	Rabbits	Thermal stress	50–400	Lactic bacteria assisted synthesis	20 and 50 mg/kg	56	25 and 50 mg of nano-Se/kg diet,ncreasing the level of only BIO from a 25 to a 50 mg/kg diet gave more improvement inthe studied parameter	[100]
	Chicken	Heat stress	100–500		0.5 mL/L	38	Weight gain, performance index, behavioral indices, MDA,SOD,immunoglobulin G, immunoglobulin M, serum total protein, albumin, alanine aminotransferase, aspartate aminotransferase, and serum creatinine concentrations increased (*P* < 0.01)	[101]
	Chicken	Oxidative stress by enrofloxacin	100	Biogenic	0.6 mg/kg	42	Activity of cellular, humoral immune response and enzymatic, non enzymatic antioxidants was significantly decreases as a result of EFX treatment	[102]
	Chicken	Oxidative stress	10–45		0.3 mg/kg	42	Highest serum IgG and IgM concentrations were recorded for non-stressed birds received nano-selenium and organic selenium	[103]
	Chicken	Cr((VI)) induced hepatic injury			0.5 mg/kg	35	Histopathological examination suggested that the liver cells of the Cr_(VI)_ poisoning group were more severely injured than the nano-Se addition group. RT-qPCR results showed that the relative expression of *ACACA* gene in the Cr_(VI)_ poisoning group was significantly increased (*P* < 0.05), while the *CPT1A* gene’s expression was significantly decreased (*P* < 0.01)	[104]
Na_2_SeO_3_	Sows	Induced heat stress (35 °C)	30–70		0.5 mg Se/kg diet	25	↓ Greatly mRNA level of *Hsp70*; ↑ mRNA level of *Hsp27*	[105]
	Sows	Induced heat stress (35 °C)	30–70		0.5 mg Se/kg diet	25	↑ Superoxide dismutase, catalase, superoxide dismutase, immunoglobulin G (IgG) and immunoglobulin A (IgA) in the serum and liver; ↓ malondialdehyde in the serum and liver	[106]

This area for the toxicological evaluation of SeNPs have mainly focused only on antioxidant system performance, body weight, and bioaccumulation in the liver, kidney and heart. There is a paucity of literature on the interaction of SeNPs with the immune system, gastrointestinal tract, immune system, or bioaccumulation in muscles and other indirect targets of Se. Due to a large surface area and small size, SeNPs and many other types of nanoparticles seem to be more reactive and show better biodistribution in organisms compared to other forms of Se. Some studies described below have examined the molecular mechanism of toxicity induced by SeNPs, as well as the comparison of acute and long-term toxicity.

Most studies that have compared the toxicity of Se and SeNPs both agree well with the lower toxicity of SeNPs. Sublethal doses of 20 nm SeNPs at doses of 0.05, 0.5, or 4 mg Se/kg body weight (BW)/d had no adverse effect on brain neurotransimeters or hematological parameters in rats compared to control and sodium selenite-treated groups group (0.5 mg Se/kg body weight/d) in a 28-day trial [35]. In similar research, low doses of SeNPs did not cause harmful effect during 48 days of treatment in rabbits. Both SeNPs and sodium selenite showed no significant changes in blood biochemistry and liver enzyme activity at a dose of 0.3 mg/kg BW. Only liver PGx and T-AOC activity were increased in Se-treated groups compared to the control group. Biochemical analysis was supported by higher *GPX-1* mRNA expression of 195% for Nano-Se and 154% for sodium selenite [36]. Higher doses of 2.0, 4.0 and 8.0 mg Se/kg body weight of SeNPs administered for 14 d caused increased body weight, increased liver enzymes (ALT, AST) and cholesterol. Histopathological findings showed lesions in the liver, kidneys, lungs and thymus gland. The presence of apoptotic cells was also observed, indicating that doses greater than 2 mg Se/kg BW induced chronic toxicity [37]. Similar findings were found in male rats treated with SeNPs at doses of 2, 4 and 8 mg Se/kg body weight for two weeks. Administration of SeNP above 4.0 mg Se/kg body weight decreased antioxidant capacities in the liver heart, and blood serum, and downregulated mRNA expression of *GPX1* and *GPX4* in the liver. The proposed mechanism of SeNPs toxicity was further demonstrated in buffalo rat liver cell lines. SeNPs at a concentration of 24 mol/L decreased cell viability and damaged antioxidant capacity. The decrease in cell viability induced by SeNPs was mainly due to apoptosis but not cell necrosis [38]. A comprehensive toxicological study showed that the 20–60 nm SeNPs and Se-methionine in supranational amounts (30 and 70 μg Se/kg BW) improved the Se accumulation in whole blood, liver and kidney in a dose-dependent manner compared to the control. At the dietary level of Se (1000 mg Se/kg BW), no improving effect of bioaccumulation in blood and tissues was observed in the case of SeNPs but not in Se-methionine form. No difference was observed between Se-methionine and SeNPs with regard to GPx activity in plasma, liver and kidneys. However, compared to Se-Met, SeNPs showed lower toxicity (LD_50_ 92.1 mg/Se/kg for Se-Met and 14.6 mg/Se/kg for SeNPs) and fewer markers of acute liver injury. A reduced accumulation of Se in dietary amounts and a higher lethal dose in mice fed SeNPs confirms the possibility of using SeNPs to avoid Se toxicity [39]. The proposed mechanism works via different absorption of Se by cells and their phase 2 response [40].

While SeNPs have shown variable toxicological outcomes, bionically or green synthesized and modified NPs have been reported which improving the effect on model animal health and reduce toxicity. The main advantage of bionic NPs appears to be the mechanism of their synthesis, which leads to the enrichment of SeNPs with bioactive compounds. Because of this ability, bionic SeNPs have unique properties. The advantages of bionic and green synthesized NPs have been well-documented in several review articles [41]. To be specific for SeNPs, the comparative study of Shakibaie et al. [53] was introduced. SeNPs (20,200 nm) were isolated from *Bacillus* sp. and orally administered to rats at doses of 2.5, 5, 10 and 20 mg Se/kg BW for 14 d. Compared to SeO_2_, bionic SeNPs showed a 26-fold lower LD_50_, while no harmful effects on the organism were observed at a lower dose [40]. Not only are bionic NPs able to reduce the toxic effect, but surface modifications make it possible to reduce the Se reactivity. κ-carrageenan-capped SeNPs (6.8 and 24.5 nm) at a dose of 2 mg/kg BW did not cause visible macroscopic or microscopic damage to major internal organs and systems in mice. However, an increased bioaccumulation of 6.8 nm SeNPs was found in liver, kidney and brain. Further experiments within the same study showed a size-dependent antioxidant activity of SeNPs, while smaller SeNPs showed a higher ability to scavenge free radicals ABTS and DPPH. These results clarified that not only the size of SeNPs might play a role in Se bioaccumulation, but their reactivity allows them to participate in biochemical interactions with organic compounds [42]. However, the vast majority of researchers have not considered the long-term toxicity of SeNPs. To illustrate, in Xiao’s study, the first experiment showed an enhancing effect of SeNPs (50 g Se/kg/d) in ApoE−/− mice in an 8-week experiment [43]. In another 24-week experiment, SeNP supplementation eliminated atherosclerotic lesions and increased antioxidant stress by inhibiting antioxidant enzymes. In addition, metabolic liver damage and hyperlipidemia have been observed. The negative effects were also size dependent, possibly due to cellular uptake. Nevertheless, the long-term toxicity of SeNPs was still lower than that of sodium selenite [44].

In general, therefore, it appears that the toxicity of SeNPs is a function of several interrelated parameters such as nanoparticle size and chemistry of the SeNP, dose, and exposure time that affect the biological response of the organism. The results of toxicological studies have shown that the main targets of the toxicity of SeNPs are not only prooxidative properties, but also their interactions with metabolic pathways and molecular signaling pathways, including apoptotic pathways, the ability of small nanoparticles to penetrate various tissues, and the organism’s ability to enzymatic transformation and eliminate Se.

## Conclusion

SeNPs and Se species have very similar mechanisms of action and toxicity. The biggest differences in their action are due to their size and different reactivity. SeNPs are more bioavailable due to their small size, and according to some studies have greater antioxidant potential. Toxicological studies indicate that they are less toxic than sodium selenite. However, in research articles dealing with chemoprotective effects, SeNPs always appear to have improving effect at lower concentrations compared to sodium selenite. These findings could implicate that the effect of SeNPs depends on the individual saturation of the selenium-treated organism.

## Data Availability

The manuscript does not contain any experimental data.

## Abbreviations

BSA: Bovine serum albumin
DNA: Deoxyribose nucleic acid
GH: Growth hormone
GSH: Glutathione
GPx: Glutathione peroxidase
GSTs: Glutathione S-transferase
IGF: Insulin growth-like factor axis
IGF-1R: Insulin growth like factor type 1 receptor
IgG: Immunoglobulin G
IgM: Immunoglobulin M
MDA: Malondialdehyde
Na_2_SeO_3_: Sodium selenite
Nm: Nano meter
NPs: Nanoparticles
Se: Selenium
Sec: Selenocysteine
Se^2−^: Selenide
SeLPs: Selenoproteins
SeMet: Selenomethionine
SeNPs: Selenium nanoparticles
Se-Yeast: Se-enriched yeast
SOD: Superoxide dismutase
Zn: Zinc
